# Symptoms of premenstrual syndrome in female migraineurs with and without menstrual migraine

**DOI:** 10.1186/s10194-018-0931-6

**Published:** 2018-10-17

**Authors:** Kjersti Grøtta Vetvik, E. Anne MacGregor, Christofer Lundqvist, Michael Bjørn Russell

**Affiliations:** 1 Head and Neck Research Group, Research Centre, Akershus University hospital, Lørenskog, Norway; 2 Department of Neurology, Akershus University Hospital, 1478 Lørenskog, Norway; 3 Centre for Neuroscience and Trauma, Blizard Institute, Barts and the London School of Medicine and Dentistry, London, UK; 4 Barts and the London NHS Trust, London, UK; 5 HØKH, Research Centre, Akershus University Hospital, Lørenskog, Norway; 6 Institute of Clinical Medicine, Campus Akershus University Hospital, University of Oslo, Oslo, Norway

**Keywords:** Migraine, Menstrual migraine, Premenstrual syndrome, Hormones, Menstruation, Menstrual cycle

## Abstract

**Background:**

Menstrual migraine (MM) and premenstrual syndrome (PMS) are two conditions linked to specific phases of the menstrual cycle. The exact pathophysiological mechanisms are not fully understood, but both conditions are hypothesized to be triggered by female sex hormones. Co-occurrence of MM and PMS is controversial. The objective of this population-based study was to compare self-assessed symptoms of PMS in female migraineurs with and without MM. A total of 237 women from the general population who self-reported migraine in at least50% of their menstruations in a screening questionnaire were invited to a clinical interview and diagnosed by a neurologist according to the International Classification of Headache Disorders II (ICHD II), including the appendix criteria for MM. All women were asked to complete a self-administered form containing 11 questions about PMS-symptoms adapted from the Diagnostic and Statistical Manual of Mental Disorders. The number of PMS symptoms was compared among migraineurs with and without MM. In addition, each participant completed the Headache Impact test (HIT-6) and Migraine Disability Assessment Score (MIDAS).

**Findings:**

A total of 193 women returned a complete PMS questionnaire, of which 67 women were excluded from the analyses due to current use of hormonal contraception (*n* = 61) or because they did not fulfil the ICHD-criteria for migraine (*n* = 6). Among the remaining 126 migraineurs, 78 had MM and 48 non-menstrually related migraine. PMS symptoms were equally frequent in migraineurs with and without MM (5.4 vs. 5.9, *p* = 0.84). Women with MM reported more migraine days/month, longer lasting migraine attacks and higher HIT-6 scores than those without MM, but MIDAS scores were similar.

**Conclusion:**

We did not find any difference in number of self-reported PMS-symptoms between migraineurs with and without MM.

## Introduction

Migraine is two to three times more prevalent in women compared to men during reproductive age [1, 2]. This female preponderance is hypothesized to result from the influence of female sex hormones on neurotransmitter systems and neuropeptide release involved in migraine pathophysiology, neuronal excitability, and underlying genetic and environmental factors [1]. In women, the incidence of migraine attacks varies across the menstrual cycle, peaking on 5 days centering on the first day of menstruation [3]. In a subset of female migraineurs, migraine attacks occur regularly and predominantly in this perimenstrual phase. The International Classification of Headache Disorders 3rd edition (ICHD 3) defines menstrual migraine (MM) as migraine attacks occurring on day − 2 to + 3 of the menstrual cycle in at least two out of three consecutive menstrual cycles [4]. Previous editions of the classification, i.e. ICHD 2 and ICHD 3 beta, restricted MM to include only migraine without aura, but the ICHD 3 provides alternative criteria for MM with aura [4–6]. MM with and without aura are further subdivided into pure menstrual migraine (PMM) and menstrually-related migraine (MRM). Women with PMM have migraine attacks occurring exclusively at menstruation, while women with MRM have additional non-menstrual attacks. For research purposes, a prospective headache diary for at least three consecutive menstrual cycles is recommended to confirm the diagnosis. More than 50% of female migraineurs report an association between migraine and menstruation, but the prevalence of MM is only about 20% among female migraineurs when the diagnostic criteria are used [7, 8].

Premenstrual syndrome (PMS) is another disorder directly linked to a specific phase of the menstrual cycle. It presents with a wide variety of physiological and psychological symptoms occurring in the luteal phase which resolve during or shortly after onset of menstruation [9–11]. A symptom-free interval between the end of menstruation and the time of ovulation is required in order to exclude other disorders [10]. The diagnosis can only be made in presence of ovulation, thus excluding women using most types of hormonal contraception. Different diagnostic criteria for PMS exist, but a common feature is the requirement that symptoms must cause significant impairment and interfere with daily functioning [9]. The diagnosis should additionally be confirmed by a symptom diary kept over at least two cycles [11]. Although up to 80% of all women report mild symptoms of PMS, only about one fourth fulfill diagnostic criteria for PMS [12, 13].

To date, few studies have looked at a possible relationship between PMS and MM and the available studies provide diverging results [14–17]. A link between PMS and MM is therefore controversial [18, 19]. The aim of the present study was to explore the association between MM and PMS by comparing self-reported PMS-symptoms in female migraineurs from the general population with and without MM without aura.

## Methods

The current paper presents results from an epidemiological study on MM from the general population and the method has been described elsewhere [7]. In January 2005, a random sample of 5000 women aged 30–34 years from the general population received a mailed screening questionnaire about migraine and the relationship of attacks to menstruation. In 2011/2012, women with self-reported migraine in at least 50% of their menstruations and < 180 headache days during the preceding year were invited to a semi-structured interview conducted by a neurologist. The interview focused on headache and migraine, migraine treatment, menstrual symptoms, and use of hormonal contraception.

Each participant was also asked to complete a self-administered questionnaire including demographic data, the impact of headache/migraine (Headache Impact test, HIT-6 and Migraine Disability Assessment Scale, MIDAS), as well as questions about PMS. The 11 PMS-questions consisted of a blinded forward-backward translation to Norwegian of the research criteria for premenstrual dysphoric syndrome (PMDD) from the *Diagnostic and Statistical Manual of Mental Disorders 4th Edition* (DSM IV) (with the possible answers yes/no [20].

Headaches were classified according to the International Classification of Headache Disorders 2nd edition (ICHD 2) including the appendix criteria for MM without aura [6]. Women with migraine, who did not fulfil these criteria, were classified as non-menstrual migraineurs (nMM). No prospective diaries were used to confirm the diagnoses.

Women were excluded from analyzes if they were currently using hormonal contraception or were not diagnosed with migraine by the neurologist.

### Statistics

The data were analyzed using SPSS version 22. χ^2^-tests and Students *t*-tests were used to compare categorical and continuous data. A p-level below 0.05 was considered significant, with the exception of one analysis with multiple tests (comparison of 11 specific items of the PMS-questionnaire) for which a Bonferroni-correction was performed and the level of significance was reduced to 0.0045 (0.05/11).

## Findings

**Fig. 1:**
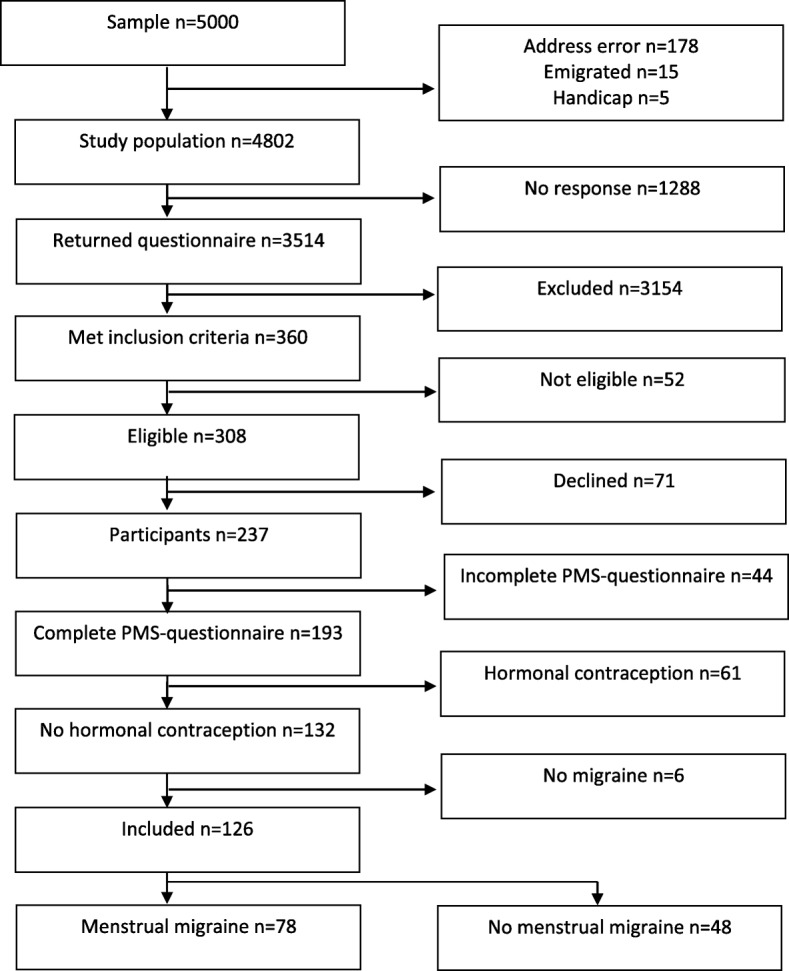
Flow chart of the study

The response rate for the questionnaire was 73%. Of 360 women reporting migraine in ≥1/2 of their menstruations, 52 were not eligible due to insufficient Norwegian language skills (*n* = 9), emigration (*n* = 4) and no reply to at least five phone calls (*n* = 39). Among the eligible, 77% (237) participated in the interview (Fig. 1).

Of the 237 women who were interviewed, 193 (81.4%) returned a complete PMS-questionnaire. Another 67 women were excluded from the analyses due to current use of hormonal contraception (*n* = 61) or because they did not fulfill the ICHD-criteria for migraine by interview (*n* = 6). Among the remaining 126 women, 78 had MM (of which six had PMM) and 48 had nMM (Fig. 1).

Women with MM reported a significantly higher number of migraine days/month, longer duration of attacks and a higher HIT-6 score than did women without MM (Table 1). Of the eight women with MM who used migraine prophylaxis, four used beta blockers, two candesartan, one onabotolinum toxin A, and one topiramate. In the nMM group, one woman was using onabotolinum toxin A.

**Table 1:** Characteristics of the participants

	Menstrual migraine (MM) *n* = 78	Non-menstrual migraine (nMM) *n* = 48	
	mean (±SD)	mean (±SD)	*p*
Migraine frequency (days/month)	4.1 (4.5)	2.2 (2.3)	0.008
Migraine pain intensity (NRS 0–10)^a^	7.9 (1.3)	7.8 (1.5)	0.27
HIT-6 score	60.4 (6.3)	57.8 (6.8)	0.035
MIDAS score	15.9 (19.3)	12.4 (16.1)	0.33
Self-reported menstrual pain (NRS 0–10)^a^	4.3 (2.9)	3.6 (2.7)	0.23
	*n* (%)	*n* (%)	
Migraine attack duration			
4- < 24 h	23 (29.5)	25 (52.1)	
24 h- < 72 h	37 (47.4)	20 (41.7)	
> 72 h	18 (23.1)	3 (6.3)	0.017
Employed	60 (76.9)	36 (75.0)	0.81
Higher education	46 (59.7)	28 (58.3)	0.88
Menorrhagia	24 (30.8)	17 (35.4)	0.59
Migraine prophylaxis	8 (10.3)	1 (2.1)	0.26

^a^Numeric Rating Scale; 0 = no pain, 10 = maximal pain

The mean number of reported PMS-symptoms among all women was 5.6 (±2.9) out of 11, with no difference between women with and without MM (5.4 vs 5.9, *p* = 0.84).The individual responses to the specific PMS-items are shown in Table 2. Women with nMM reported more premenstrual affective symptoms (question 1–2), but after correction for multiple testing, the differences were not statistically significant.

**Table 2:** Responses to the specific questions of PMS-symptoms from DSM IV among women with and without MM

“Do you have any of the following symptoms during the last week before onset of menstruation, which disappear within few days after onset of the menstrual bleeding?”		Menstrualmigraine (MM) *n* = 78		Non-menstrual migraine (nMM) *n* = 48		
		%	*n*	%	*n*	*p*
*1*	*Markedly depressed mood, feelings of hopelessness, or self-deprecating thoughts*	38.5	30	60.4	29	0.02
*2*	*Marked anxiety, tension, feelings of being “keyed up” or “on edge”*	30.8	24	52.1	25	0.02
*3*	*Marked affective lability (*e.g.*, feeling suddenly sad or tearful or increased sensitivity to rejection)*	62.8	49	66.7	32	0.66
*4*	*Persistent and marked anger or irritability or increased interpersonal conflicts*	57.1	44	47.9	23	0.31
*5*	*Decreased interest in usual activities (*e.g.*, work, school, friends, hobbies)*	39.7	31	31.3	15	0.34
*6*	*Subjective sense of difficulty in concentrating*	33.3	26	37.0	17	0.68
*7*	*Lethargy, easy fatigability, or marked lack of energy*	67.9	53	76.6	36	0.30
*8*	*Marked change in appetite, overeating, or specific food cravings*	59.0	46	62.5	30	0.69
*9*	*Hypersomnia or insomnia*	42.3	33	43.8	21	0.87
*10*	*A subjective sense of being overwhelmed or out of control*	11.7	9	18.8	9	0.27
*11*	*Other physical symptoms, such as breast tenderness or swelling, headaches, joint or muscle pain, a sensation of “bloating,” weight gain*	94.9	74	91.7	44	0.47

## Discussion

In this study, women with MM did not report more PMS symptoms than did women with nMM. A significantly higher HIT-6 score, more migraine days/month and longer duration of migraine attacks was reported by women with MM, while MIDAS scores were similar between the groups.

Our results must be interpreted with caution for several reasons. Firstly, we used the DSM-IV criteria, which mainly emphasize the psychological and affective aspects of PMS. Only one out of the 11 questions relates to physiological symptoms. Other definitions of PMS, such as those from the International Classification of Disorders 10th edition (ICD − 10), the International Society for Premenstrual Disorders (ISPMD), and the Royal College of Obstetricians and Gynaecologists (RCOG) equate physical and psychological symptoms [11, 21, 22]. Our study does therefore mainly detect psychological symptoms and one can only speculate whether application of other diagnostic criteria would have provided different results. Secondly, our PMS-data are limited due to retrospective self-reporting, the lack of information about the severity of the different symptoms, and whether they interfere with daily living. We can consequently only conclude about occurrence of premenstrual symptoms, but not about the prevalence of PMS in this population, or the severity and the degree of interference with daily functioning. This was, however, the case for all participants in both groups and cannot explain the lack of difference between the women with and without MM.

The MM diagnoses were based on clinical interviews and not confirmed by prospective headache and menstruation diaries. We have previously validated the clinical interview diagnoses against the women’s headache diaries and found a good chance-corrected agreement rate (Kappa 0.62) [23]. As the sample was chosen based on self-reported migraine in at least 50% of all menstruations, it may not be representative of the general female migraine population. This might have contributed to the lack of difference between women with and without a current diagnosis of MM.

None of the women used antidepressant drugs as migraine prophylaxis. We have no information about possible psychiatric comorbidity and use of antidepressant drugs for other reasons than migraine, e.g. the use of SSRI for management of PMS. The participants were aged 36–40 years and our findings may not be valid to women at other ages. Finally, this cross-sectional design is not optimal for studying this topic. In the future, longitudinal studies with prospective symptom records are recommended.

Previous studies have reported an increased risk of migraine in women with PMS [14, 24], but few studies have specifically addressed occurrence of PMS in women with MM. We identified only four studies presenting figures for both MM and PMS, two of which were population-based.

A Swedish population-based study including 728 women aged 40–74 years, reported a significant increased risk of migraine in women with PMS, but no difference in occurrence of PMS among migraineurs with and without MM (16% vs 10%, *p* = 0.55) [14]. The PMS-diagnoses were based on the DSM-criteria, while MM was defined more strictly than the current ICHD-criteria (≥75% of all attacks should occur on day 1 ± 2 of the menstrual cycle). Both diagnoses were retrospectively assessed.

In contrast, a Taiwanese population-based study including 1436 women aged 40–55 years reported a significantly higher prevalence of self-reported MM among migraineurs with PMS compared to migraineurs without PMS (57.7% vs. 38.9%, *p* < 0.01) [16]. The MM diagnoses were not criteria-based, but retrospectively defined as “migraine attacks occurring more frequently two days before or during menses”, without any requirement for frequency of attacks in relation to menstruation. PMS was retrospectively diagnosed by self-administered questionnaires based on the International Classification of Disorders 10th edition (ICD-10) with requirement of at least one of seven specific physical or mood symptoms occurring in a cyclic fashion.

Direct comparisons to other studies are challenging because these studies are based on selected clinic populations [15, 17]. An Italian study including 64 women attending the Psychobiology of Reproduction Unit of the Department of Obstetrics and Gynecology in Modena, reports that more than half of women with PMM (14/22) and one third of women with MRM (4/12) had PMS [15]. Another report from Japan including 83 women with premenstrual dysphoric disorder, defined as a condition where the symptoms are at the extreme end of the psychological spectrum, presents a very high prevalence of migraine (68.7%). More than 90% of all women with migraine without aura had MM in that sample [17].

The diverging results across studies may thus be explained by methodological differences, such as differences in definitions of PMS and MM, assessment methods (prospective ratings vs. retrospective reports of symptoms) and study populations.

Of interest is the similarity between PMS-symptoms and premonitory symptoms of migraine, which occur in up to 80% of all migraineurs 2–48 h before onset of migraine attacks [25]. Examples of such symptoms are food craving, mood changes, and lethargy which are hypothesized to be driven by pre-ictal changes in hypothalamic and dopaminergic activity [26, 27]. Headache is also included in the spectrum of physiological symptom of PMS [10, 22]. Hence, there is an overlap not only concerning the timing of PMS and MM, but also an overlap of symptoms. Women who regularly experience MM attacks may also regularly experience premonitory migraine symptoms mimicking PMS. This could perhaps explain the high prevalence of PMS detected in some MM-populations using other diagnostic criteria for both MM and PMS than we did [15, 16]. Although women with MM in our study reported a higher impact of migraine in terms of higher HIT-scores, more migraine days and longer duration of attacks, PMS-symptoms were equally frequent in women with and without MM. This might suggest different mechanisms underlying MM and PMS.

Similar for both conditions is the cyclic occurrence of symptoms in relation to specific phases of the menstrual cycle. An interaction between the neuroendocrine system and an oversensitivity for hormonal changes, with abnormal response to physiological hormonal changes are hypothesized to underlie both PMS and MM [1, 13, 28, 29].

Most studies do not demonstrate any abnormalities in blood levels of estrogen and progesterone in women with PMS or MM compared to healthy women [28, 30]. However, in PMS an association with rate of change in progesterone levels has been reported [31]. In contrast, a faster decline in estrogen-levels in the luteal phase has been reported in female migraineurs [28]. This ‘estrogen-withdrawal’ mechanism for MM is independent of both progesterone and ovulation [32]. There are many different theories regarding the pathophysiology of PMS-symptoms. The main theory suggests the latter is a consequence of presence of progesterone and perhaps its withdrawal and that it can therefore only occur in ovulatory cycles [13, 29]. In addition, the involvement of a hypo-serotoninergic state has been suggested. The management of both conditions is directed towards these different mechanisms; in PMS it is aimed at modulation of serotonin (SSRIs) and suppression of ovulation [11, 22, 29], while the main target in MM is prevention of estrogen-withdrawal in the late luteal phase [33]. The time frames of occurrence are only partly overlapping as PMS-symptoms start before the typical onset of MM. In addition, PMS can only be diagnosed in ovulating women and suppression of ovulation results in major reduction or elimination of PMS-symptoms. In contrast, MM occurs in situations with inhibited ovulation as long as estrogen-withdrawal occurs [32].

## Conclusion

Women with MM from this general population did not report more PMS-symptoms than did women with nMM. Although both conditions are linked to specific menstrual cycle phases with symptoms likely to be caused by changes in female sex hormones, it is unlikely that they share a common pathophysiological pathway.

Future studies should include prospective symptom recording of both premonitory migraine symptoms and PMS-symptoms as well as the use of established and generally accepted diagnostic criteria. Collection of daily hormones could additionally contribute a better understanding of hormonal changes in both conditions. As this study mainly covers the psychological aspects of PMS, future studies should also explore the physical dimensions.

## Abbreviations

MM: menstrual migraine
MRM: menstrually-related migraine
nMM: non menstrually-related migraine
PMM: pure menstrual migraine
PMS: premenstrual syndrome
